# Rodent Gut Bacteria Coexisting with an Insect Gut Virus in Tapeworm Parasitic Cysts: Metagenomic Evidence of Microbial Selection in Extra-Intestinal Clinical Niches

**DOI:** 10.3390/microorganisms12061130

**Published:** 2024-05-31

**Authors:** Amro Ammar, Vaidhvi Singh, Sanja Ilic, Fnu Samiksha, Antoinette Marsh, Alexander Rodriguez-Palacios

**Affiliations:** 1 Division of Gastroenterology and Liver Disease, Case Western Reserve University School of Medicine, Cleveland, OH 44106, USA; axa2015@case.edu (A.A.); vxs390@case.edu (V.S.); 2 Digestive Health Research Institute, Case Western Reserve University School of Medicine, Cleveland, OH 44106, USA; 3 Department of Human Sciences, Human Nutrition and Food Microbiology, The Ohio State University, Columbus, OH 43210, USA; ilic.2@osu.edu; 4 Department of Cancer Biology, Learner Research Institute, Cleveland Clinic, Cleveland, OH 44106, USA; samiksh@ccf.org; 5 The Veterinary Medical Center Diagnostic Parasitology, Department of Veterinary Preventive Medicine, College of Veterinary Medicine, The Ohio State University, Columbus, OH 43210, USA; marsh.2061@osu.edu; 6 Department of Molecular Biology and Microbiology, Case Western Reserve University School of Medicine, Cleveland, OH 44106, USA; 7 University Hospitals Research and Education Institute, University Hospitals Cleveland Medical Center, Cleveland, OH 44106, USA

**Keywords:** *Bacteroidetes*, *Mythimna unipuncta granulovirus* A, *Hydatigera taeniaeformis*, cysticercosis, *Parabacteroides distasonis*, *Klebsiella*, cavernous fistulous tracts, CavFT, metacestode, *P. cavitamuralis*

## Abstract

In medicine, parasitic cysts (e.g., brain cysticerci) are believed to be sterile, and are primarily treated with antiparasitic medications, not antibiotics, which could prevent abscess formation and localized inflammation. This study quantified the microbial composition of parasitic cysts in a wild rodent, using multi-kingdom metagenomics to comprehensively assess if parasitic cysts are sterile, and further understand gut microbial translocation and adaptation in wildlife confined environments, outside the gut. Analysis was conducted on DNA from two hepatic parasitic cysts from a feline tapeworm, *Hydatigera (Taenia) taeniaeformis*, affecting a wild vole mouse (*Microtus pennsylvanicus*), and from feces, liver and peritoneal fluid of this and two other concurrent individual wild voles trapped during pest control in one of our university research vegetable gardens. Bacterial metagenomics revealed the presence of gut commensal/opportunistic species, *Parabacteroides distasonis, Bacteroides (Bacteroidota)*; *Klebsiella variicola, E. coli (Enterobacteriaceae); Enterococcus faecium* and *Lactobacillus acidophilus (Bacillota)* inhabiting the cysts, and peritoneal fluid. Remarkably, viral metagenomics revealed various murine viral species, and unexpectedly, a virus from the insect armyworm moth (*Pseudaletia/Mythimna unipuncta*), known as *Mythimna unipuncta granulovirus* A (MyunGV-A), in both cysts, and in one fecal and one peritoneal sample from the other non-cyst voles, indicating the survival and adaption potential of the insect virus in voles. Metagenomics also revealed a significantly lower probability of fungal detection in cysts compared to that in peritoneal fluid/feces (*p* < 0.05), with single taxon detection in each cyst (*Malassezia* and *Pseudophaeomoniella oleicola*). The peritoneal fluid had the highest probability for fungi. In conclusion, metagenomics revealed that bacteria/viruses/fungi coexist within parasitic cysts supporting the potential therapeutic benefits of antibiotics in cystic diseases, and in inflammatory microniches of chronic diseases, such as Crohn’s disease gut wall cavitating micropathologies, from which we recently isolated similar synergistic pathogenic *Bacteroidota* and *Enterobacteriaceae*, and *Bacillota*.

## 1. Introduction

The microbial communities, with their diversity and interactions, continue to intrigue scientists due to the unforeseen complexities of symbiosis in many ecosystems [1,2], including spatially-confined niches such as within parasitic cysts. The interplay of microbial populations is critical in maintaining homeostasis and health in the mammalian gut ecology. Therefore, the study of these microbial communities could help us determine the influences of these dynamic relationships in restricted ecological niches [3,4]. An understanding of the features and evasion mechanisms of host immunity in confined microbial niches, such as within gut wall-associated cavitating fistulous tract (CavFT) micropathologies in Crohn’s disease (CD) [5,6] could help improve current therapies. Of note, we recently determined through genome sequence analysis of bacteria that CavFT have niche-specific genomic exchange across species within *Bacteroidota* (*Bacteroidetes*), but not *Enterobacteriaceae*, which could favor silent commensalism and immune evasion in individuals susceptible to disease and succinate-producing *Bacteroidota* such as *Parabacteroides distasonis* and related *Bacteroides* [6,7,8,9].

The relevance of cysticerci in medicine is associated with the diseases that cysticerci may induce as tapeworm in the intermediate stages travel as larval parasites through the body and stop to enter into their seemingly quiescent cystic stage, where they become space-occupying masses that gradually develop unnoticed by affected individuals, and which could drag gut bacteria on integumentary micro-cavitations, as seen with electron microscopy in tapeworms [10]. Microbes carried on the surface of the gut, could also be primarily derived from ingested and bypassing foods within the gut, which could then be internalized or ‘translocated’ as parasitic larvae migrate. Migratory parasites entering in contact with tissues would trigger host immunity in poorly understood ways which could presumably stress and drive the selection of microbial communities that successfully survive evading the immune system. How these communities are assembled in areas distant from the gut where there is a narrow range of nutrient sources and stressors, and where there is no physical removal of bacteria by peristalsis, remains unknown.

Commonly, adult parasitic tapeworms in domestic animals live in carnivorous species, including humans, which produce eggs and then develop cysts within tissues (e.g. brain cysticerci) and the peritoneal cavity as the larva migrate throughout the host after ingestion and activation in the intestinal tract [11,12,13]. *Echinococcus granulosus* and other parasites alike [12], including *Taenia* and *Hydatigera*, are some of the many pathogenic parasites that seem to facilitate intricate interactions within their intermediary hosts during migration at the larva stage [12]. Assessing such migratory and selection process of bacteria from the gut is critical to help us determine to what extent parasitic cysts contain microbial species that could be treated medically. Brain cysticerci in humans is especially important in children who are almost always affected by one cysticercus [14]. Thus, the study of parasitic cysts in rodents [15] provides an attractive model for studying microbial survival and symbiosis away from the gut and in conditions of limited nutrient (dietary and fecal) availability.

Herein, we report the results from a metagenomics community composition analysis in various tissues from three wild voles obtained as discarded dead individuals in a location that underwent pest control, one of which was affected with two extra-hepatic parasitic cysts that resembled the appearance of *Echinococcus granulosus* cysts (present in 80.5% of affected humans [14]; dimensions: 10 and 12 mm diameter), but they were confirmed as the tapeworm genus *Hydatigera* (formerly *Taeniea*). The purpose of this study was to assess and report the metagenomic community composition of these parasitic cysts in the context of other organs (feces, liver and peritoneal fluid) to catalogue the bacterial communities that may translocate within parasitic larval stages, and thrive outside of the typical gut milieu, in parasitic cysts.

## 2. Methods

### 2.1. Animals and Location

Voles are common prolific inhabitants in rural and urban-peri rural areas in Ohio, USA. The animal samples correspond to three (n = 3) vole (*Microtus pennsylvanicus*) mice obtained dead (convenience sampling) as disposal pest control material after a routine mouse pest control day at the Ohio Agricultural Research and Development Center farm vegetable garden in 2011. Animals were trapped in a single location using snap mouse traps, which are classified as IACUC-approved traps for mouse pest control in the USA. Studies have shown that animals fall rapidly unconscious when hit in the head/neck region by a snap trap [16]. No IACUC approval was required for this pest-control based sampling study of ‘discarded materials’ or for the collection and freezing of tissues under such circumstances at the time. According to the ‘2016 Guidelines of the American Society of Mammalogists for the use of wild mammals in research and education’, issued by the Animal Care and Use Committee of the American Society of Mammalogists [17], snap traps are considered humane and a preferred method compared to drowning and glue and pitfall ethanol traps because snap traps ‘inflict the least trauma and result in a clean, effective kill’ [17]. No other animals were used in this study.

### 2.2. Identification of the Parasitic Cysts

The frozen cystic-like structure and the cyst fluid DNA samples were sent overnight to the Diagnostic Veterinary Parasitology Laboratory, at the Veterinary Medical Center, The Ohio State University. The cyst was thawed briefly, extraneous host tissue removed from the cream/white colored cyst using small forceps and a needle. The cyst was placed on a microscope, covered with a coverslip and pressure applied to flatten the structure for photo microscopy. Images were captured using an Olympus BX41 with cellSens software (standard 1.18, Olympus, USA).

### 2.3. Visualization and Anaerobic Culture of Fluid from the Parasitic Cysts

To help determine whether the cysts were harboring live bacteria, we visualized the liquid in phase contrast medium using 1000× magnification, and also cultured [18] the fluid by spread-plating 20 microliters onto a pre-reduced tryptic soy 5% defibrinated sheep-blood agar (80% N–10% H–10% CO_2_, at 37 °C; Thermo Fisher Scientific, USA) using a variable-atmosphere anaerobic Whitley workstation A85 (540 plate capacity; Microbiology International, Inc., USA) as described [6,9]. Individual colonies were sub-cultured and purified in the same agar and then immediately identified using matrix-assisted laser desorption ionization–time of flight [MALDI-TOF] mass spectrometry) and banked in pre-reduced brain heart infusion broth with 7% dimethyl sulfoxide [6,9].

### 2.4. DNA Extraction and Metagenomics

DNA extraction was conducted using the DNAeasy Qiagen kit (USA), while the DNA was quantified using the GloMax Plate Reader System (Promega, USA) using the QuantiFluor^®^ dsDNA System (Promega) chemistry. Samples were submitted for metagenomic analysis to a third party (CosmoID, USA), which has validated methods and software [19,20] for library preparation, sequencing and cloud-based computing for data analysis [21].

### 2.5. Library Preparation and Sequencing

DNA libraries were prepared using the Nextera XT DNA Library Preparation Kit (Illumina, MO, USA) and IDT Unique Dual Indexes with a total DNA input of 1 ng. Genomic DNA was fragmented using a proportional amount of Illumina Nextera XT fragmentation enzyme. Unique dual indexes were added to each sample followed by 12 cycles of PCR to construct libraries. DNA libraries were purified using AMpure magnetic beads (Beckman Coulter, USA) and eluted in QIAGEN EB buffer. DNA libraries were quantified using a Qubit 4 fluorometer and Qubit™ dsDNA HS Assay Kit (USA, Cat. Q32851). Libraries were then sequenced on an Illumina HiSeq X platform 2 × 150 bp.

### 2.6. Bioinformatics Analysis and Metagenome Classification

The system utilizes a high-performance data-mining k-mer algorithm that rapidly disambiguates millions of short sequence reads into the discrete genomes engendering the particular sequences. The methodology employed in this study uses the CosmosID-HUB for the fast and precise metagenomic analysis of microbiome data [20,21,22], incorporating detection capabilities at the strain level across multiple kingdoms, along with antimicrobial resistance/virulence factors (AMR/VF) and functional analysis within a singular processing framework, which has been shown recently to perform well compared to other pipelines [19,20]. Herein, we solely report the microbial community composition since the interpretation of functional data for *Bacteroidota* has some limitations and complexity that we recently determined and which are under investigation [8]. The complete documentation for the analysis [21] is available at https://docs.cosmosid.com/docs/methods (accessed on 20 March 2024). This analysis platform is powered by three core components, a genBook database, a Kepler algorithm and machine learning filters. The GenBook is a meticulously curated Multi-Kingdom Reference Database featuring over 180,000 genomes and gene sequences from bacteria, fungi, viruses, phages and protists. Its curation process is designed to enhance sensitivity by reducing redundancy and ensuring homogeneity, particularly in densely populated clades such as *Staphylococcus aureus*. The database universal curation approach allows for consistent analysis across various sample types within a project, ensuring accuracy through genome quality control and minimizing false positives. The Kepler Algorithm is a patented, k-mer based algorithm that offers efficient and highly accurate profiling. It utilizes unique and shared k-mers across the phylogenetic tree for precise near-neighbor placement, ensuring these k-mers are phylogenetically stable and do not overlap with mobile genetic elements or the human genome. This approach, coupled with GenBook phylogenetic ontology, permits accurate differentiation down to the strain level. Lastly, the system uses Machine Learning Filters within the analysis pipeline, which is enhanced by machine learning algorithms trained on over 10,000 samples, allowing for the distinction between genuine signals and background noise. This maintains high sensitivity and precision, as evidenced by superior F1 scores in benchmarks and community challenges.

For metagenomic analysis, whole genome shotgun sequencing data (in fastq or fasta formats) is used. Paired-end files may be combined for analysis if uploaded simultaneously. Following sample upload, the CosmosID-HUB automatically processes and generates detailed reports, including tables and visualizations of genome and gene databases, covering bacteria, fungi, protists, viruses, respiratory viruses, antimicrobial resistance and virulence factors, which are shown in this report. As for performance evaluation, studies have validated CosmosID pipeline for detection accuracy and resolution [21]. The system demonstrates exceptional identification accuracy across all taxonomic levels in benchmark datasets, significantly outperforming other tools, especially in sub-species and strain-level classification.

The metagenomics pipeline has two separable comparators, the first consists of a pre-computation phase for reference databases and the second is a per-sample computation [21]. The input to the pre-computation phase are databases of reference genomes, virulence markers and antimicrobial resistance markers that are continuously curated and added to an updated taxon database. The output of the pre-computational phase is a phylogeny tree of microbes, together with sets of variable length k-mer fingerprints (biomarkers) uniquely associated with distinct branches and leaves of the tree.

The second per-sample computational phase searches the hundreds of millions of short sequence reads, or alternatively contigs from draft de novo assemblies, against the fingerprint sets. This query enables the sensitive yet highly precise detection and taxonomic classification of microbial NGS reads. The resulting statistics are analyzed to return the fine-grain taxonomic and relative abundance estimates for the microbial NGS datasets. To exclude false positive identifications, the results are filtered using a filtering threshold derived from internal statistical scores that are determined by analyzing a large number of diverse metagenomes [21].

### 2.7. Metacestode DNA Extraction and Sequencing

Genomic DNA was extracted using DNeasy Blood & Tissue Kit (Qiagen, USA) following the manufacturer’s instructions for tissues with a slight modification. During the proteinase K digestion, the sample was continuously rotated at 56 °C for 45 min. The mitochondrial 12S rRNA gene region was targeted using 20 to 45 ng of genomic DNA per reaction along with the Applied Biosystem Power SYBR Green PCR Master Mix and previously described primers, Cest F: 5′ AGTCTATGTGCTGCTTAT 3′ and Cest R: 5′ CCTTGTTACGACTTACCT 3′ [23]. Cycling consisted of 95 °C for 2 min followed by 50 cycles of 95 °C for 15 s, 45 °C for 30 s and 60 °C for 1 min on an Applied Biosystems StepOne Instrument (MI, USA). Control DNA of *Echinococcus granulosus*, *E. multilocularis* and *Taenia* sp. Was provided by Kamilyah R. Miller (Kansas State University). The amplicon obtained from the cystic structure DNA from six different reactions was pooled and purified using QIAquick PCR Purification Kit (USA) spin columns. The purified product and primers were submitted to Genewiz for DNA sequencing. Two replicate experiments representing both forward and reverse were used to construct the consensus sequence. The 176 base-pair DNA sequence was compared to published sequences using a nucleotide Blastn search (https://www.ncbi.nlm.nih.gov/, accessed on 1 May 2024). The resulting DNA sequence was submitted to GenBank accession PP477764.

### 2.8. Statistics

This report is primarily descriptive because the number of animals and samples tested were limited. Univariate analysis of metagenomic community composition and frequency statistics (presence/absence) for species of interest across the samples [24] was conducted to determine if findings were random or significantly different from random. For this purpose, we used Fisher’s exact, or Chi-square statistics using GraphPad (v10.2.1) depending on the number of observations in each cell in 2 × n tables. Statistical significance for expected vs. observed was held at *p* < 0.05.

### 2.9. Data Availability

The metagenomic sequences and fastq files have been deposited in NCBI GenBank under the BioProject number PRJNA1053337. Entitled ‘gut microbiome that evades host immunity in wild rodents (vole) and parasitic cysts’, this project has 11 associated BioSamples and Sequence Read Archive (SRA) numbers for sharing with the scientific community under submission SUB14073752, and accessions SRR27223102 through SRR27223112, released on 18 April 2024. 

## 3. Results

### 3.1. Nucleotide Sequence Analysis of Metacestode Reveals Hydatigera taeniaeformis

**Figure 1:**
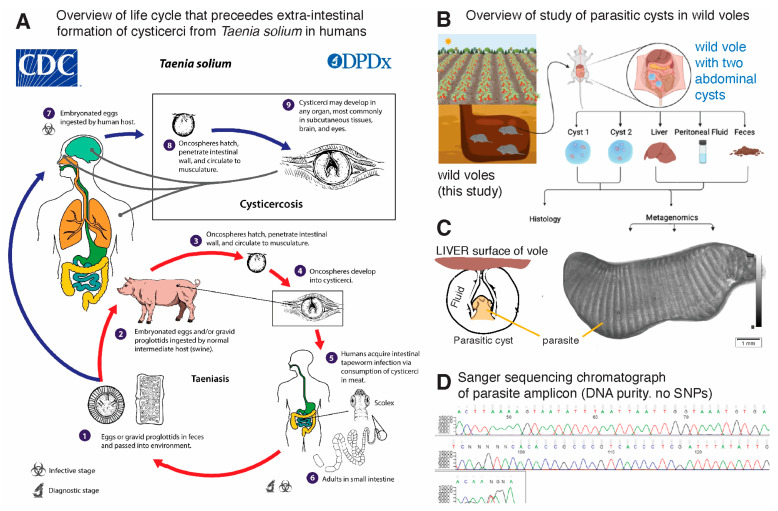
Overview of the rodents (voles) tested in this study and identification of the hepatic parasitic cyst as the larval stage of *Hydatigera taeniaeformis* tapeworm. (**A**) Contextualization of the clinical and ecological relevance of human cysticercosis, exemplified with *Taenia solium*. (CDC public domain image). (**B**) Samples collected from the voles in this report. (**C**) Schematic of origin of samples within metacestode (parasitic-cystic structure) for analysis and photomicrograph, illustrating distinctive microscopic segmentation of the parasite as indicative of an immature strobilocercus metacestode. Not detailed: the parasite lacks visualized hooks and the overall size suggests that this cyst-like structure is an immature strobilocercus. (**D**) Chromatogram after Sanger sequencing used for tapeworm identification as *Hydatigera taeniaeformis* illustrates pure DNA and homogeneous amplicon in the cyst samples tested in this study.

An overview of the cysts, fecal, peritoneal fluid and liver samples collected from three voles and processed in this study can be found in Figure 1A–D. Although initial environmental assumptions suggested that *Echinococcus* sp. was the most likely parasite (e.g., owing to the presence of coyotes and other carnivores on the farm where the vegetable garden was implemented), DNA amplification and sanger sequencing results revealed that the parasite metacestode was *Hydatigera taeniaeformis*, which forms a strobilocercus as its metacestode stage in the intermediate rodent host. The NCBI Blastn search showed the greatest percent identity (99%) to *Hydatigera* sp. (Genbank LC008533.1). Microscopic examination of the parasite confirmed that the segmentation patterns observed on the surface of the organism are suggestive of an immature strobilocercus metacestode stage which was discernable on the photomicrographs using the magnification and dorsal ventral flattening of the cyst. The lack of hooks and size suggests that this cyst-like structure is an immature strobilocercus.

### 3.2. Visualization and Cultivation of Fluid Yielded Enterococcus faecium

Notably, we were able to visualize the presence of highly mobile bacteria-like structures in the fluid examined under contrast-phase microscopy and visualized a complex array of gram-positive and gram-negative bacteria. However, also of interest, cultivation of the cysticercus fluid only revealed the presence of pure colonies of *Enterococcus faecium*, which were identified using MALDI-TOF. Although gram-staining of biological samples did not resemble the textbook gram-stain description of microbes isolated on agar surfaces, the isolation of pure *Enterococcus* on the agar (typically gram-positive cocci), and not of other bacteria, indicates that the cohabitation of multiple bacteria in a spatially-confined nutrient-depleted biological niches, such as the cysts, could be rendering *Enterococcus* species more symbiotic with other community members, instead of being inhibitory once it is growing on an artificial nutrient-rich medium such as 5% sheep blood TSA plates as we previously documented for a fecal *Enterococcus* strain against a co-inhabitant *Lactobacillus* in the intestinal tract in a mouse model of Crohn’s disease [25]. Metagenomic analyses were therein pursued to better characterize the non-cultivable species in the cysts.

### 3.3. Metagenomics of the Cystic Fluid Revealed Gut Commensal/Opportunistic Bacteria

**Figure 2:**
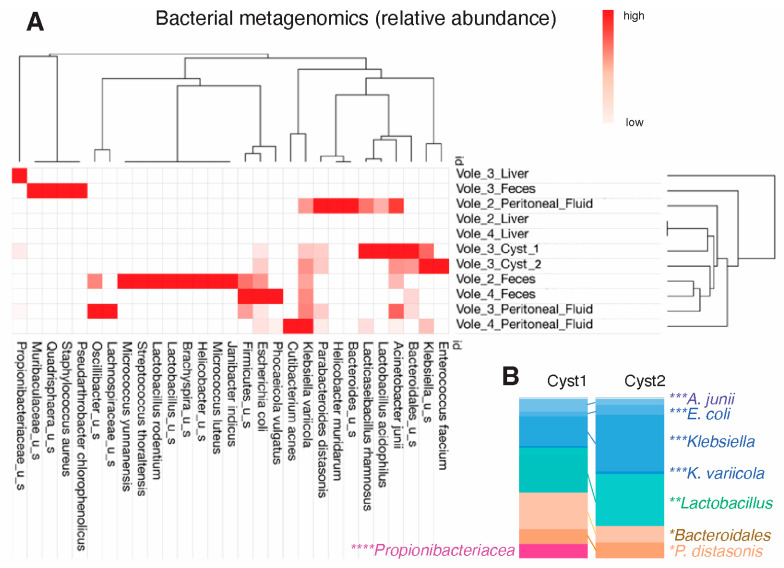
Metagenomic analysis identifies reproducible commensal *Bacteroidota* and opportunistic *Pseudomonadota* (*Enterobacteriaceae*) in the parasitic cysts. (**A**) Relative abundance across samples. (**B**) Comparison of bacteria in both cysts demonstrates consistent pattern of abundance among similar bacteria, including *K. variicola*, *P. distasonis* and *Bacteroidales.* Asterisks represent color coded species of distinct phyla; * Bacteroidota (formerly Bacteroidetes); ** Bacillota (Firmucutes); *** Pseudomonadota (Proteobacteria); **** Actinomycetota (Actinobacteria).

Metagenomic analysis revealed a relatively simple bacterial community inside the two cysts, demonstrating that these symbiotic bacteria could avoid the immune system and flourish over time in a nutrient-depleted lesion. At the species level, *Klebsiella variicola* comprised 18.34% and 35.48% of the total bacterial population in cysts 1 and 2, respectively, followed by *Enterococcus faecium* in cyst 2 (32.59%). *K. variicola* was also highly abundant in the peritoneal fluid samples of vole 2 (33.34%), vole 3 (39.86%) and vole 4 (74.9%) and in the feces samples of vole 2 (29.66%) and vole 4 (37.96%). In contrast, the vole 3 liver was entirely inhabited by *Propionibacteriaceae,* which only constituted 8.95% of cyst 1 (Figure 2A,B).

*Bacteroidales* amassed an abundance of 23.29% in cyst 1 and 10.28% in cyst 2. At the species level, cysts 1 and 2 had a very comparable abundance of *Parabacteroides distasonis* with 9.08% and 9.88%, respectively. The analysis of peritoneal fluid revealed *P. distasonis* as the most prevalent bacteria (42.39%) in vole 2, and as a highly abundant bacteria in vole 3 (9.36%). Of note, vole 3 was inhabited by *Quadrisphaera* sp. DD2A (44.69%). The latter finding is of notoriety, since the DNA of fecal samples in this study did not reveal a large number of bacterial taxonomic units, as expected from other studies we have conducted on human colonoscopy content and in mice [7,25] with the same methodology or using 16S rRNA microbiome studies [26,27]. This finding could be attributed to a completely different gut microbiome in these wild animals who inhabit subterraneous environments and have different diets. Repeated testing confirmed the limited detection of OTUs in feces in this study.

### 3.4. Identification of Insect Virus Outside Its Natural Habitat

For the first time, the presence of a virus, *Mythimna unipuncta granulovirus* A (MyunGV-A), which was adapted within the insect armyworm *Mythimna unipuncta* [28] (which feeds on crops, including corn [29]), was detected using metagenomic analysis outside of its natural insect habitat, inside parasitic cysts within voles. MyunGV-A naturally infects and replicates within the larvae of the armyworm moth, *Mythimna unipuncta*, primarily within the cells of the midgut epithelium.

**Figure 3:**
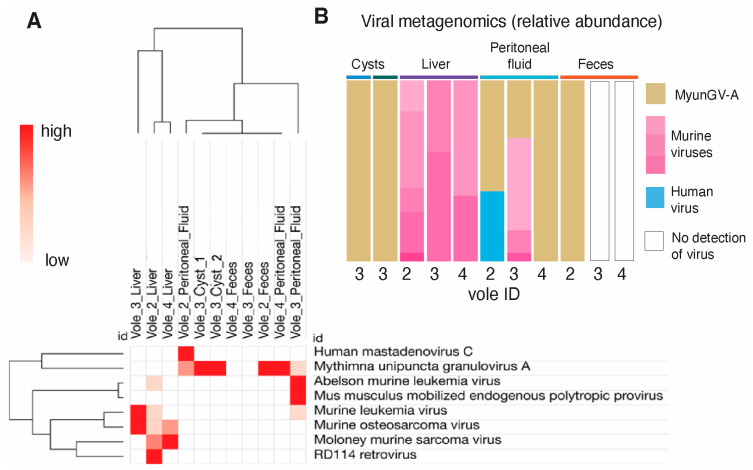
Metagenomic analysis of viruses revealed MyunGV-A in *Hydatigera* larval cysts and peritoneal fluid of wild voles. (**A**) Hierarchical clustering of samples based on abundance. Euclidean distances. (**B**) Abundance bar plot. MyunGV-A in both cysts was 100%, with similar findings in the peritoneal fluid and feces of other voles.

Our study detected the presence of MyunGV-A in high abundance, suggesting that the virus may be thriving inside the cyst through cohabitation with the bacteria, and the absence of other viruses that were identified in the liver, peritoneum and feces of the other voles. MyunGV-A was found to be the only viral species in both cysts, while it was combined with human mastadenovirus C in vole 2 peritoneal fluid (61.19%; Figure 3A,B). In vole 4 peritoneal fluid and vole 2 feces, the MyunGV-A relative abundance was 100%, since no other viruses were detected. The liver samples were mostly inhabited by Moloney murine sarcoma virus and Murine osteosarcoma virus, which are expected infectious viruses of rodents. In the peritoneal fluid of vole 3, the Abelson murine leukemia virus was the most prevalent (51.19%), followed by MyunGV-A (31.72%). This discovery indicates that MyunGV-A has probably adapted to voles, the parasite, or to the gut microbiome of mice, or that the microbiome provides metabolites that enable the virus to colonize other species (mouse or hydatigera), raising questions about the role of newly adapted, or transiently infecting baculoviruses in rodents and the implications for both the parasite and its mammalian host.

### 3.5. Metagenomics Suggests Lower Probability of Fungal Detection in the Cysts

A binary analysis of the presence or absence of fungal DNA in the samples, regardless of the species identified, revealed that the animals had a significantly lower probability of fungal detection in the cysts compared to other samples (peritoneal fluid, *p* < 0.05; feces, *p* < 0.05), with single taxon detection in each cyst for *Malassezia*, and *Pseudophaeomoniella oleicola*. Notably, the samples with a higher probability of fungal taxa were the peritoneal fluids. In summary, of statistical relevance, results from metagenomic studies of 140 other samples corresponding to concurrent experiments in our laboratory, submitted in separate batches to CosmosID, did not yield the same insect virus we detected in the cysts, further supporting that our findings do not represent reagent contamination.

## 4. Discussion

**Figure 4:**
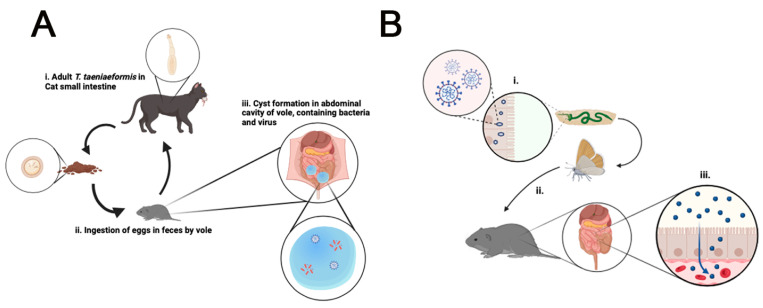
Overview of *Hydatigera taeniaeformis* lifecyle and metacestode larval cystic stages in rodents and our theory of how MyunGV-A viral DNA could reach and co-adapt with bacteria in tapeworm cysts. (**A**) Tapeworm *H. taeniaeformis* responsible for cyst formation in the peritoneal cavity in rodents may be conducive for the retention and replication of MyunGV-A and bacteria. The cysts may offer protection against external factors and create a microenvironment suitable for these microorganisms, facilitating the evasion of the immune system. (**B**) Theory for acquisition of the insect virus by voles is presumed to have occurred indirectly through dietary sources e.g., ingestion of insects or vegetation carrying MyunGV-A contaminants. Steps in proposed cycle: i., MyunGV-A replicates in the midgut epithelial cells of armyworms; ii., vole consumes armyworm moth; iii., MyunGV-A is absorbed via the brush border of the intestinal epithelial cells and into the bloodstream, disseminating to the cysts, or the virus and gut bacteria (*Bacteroidota*, *Enterobacteriaceae*, *Lactobacillus*) follow the *H. taeniaeformis* larva as it migrates through the gut wall to extra-intestinal tissues to start the cystic stage of the tapeworm.

Herein, we report the results from a metagenomics community composition analysis of various tissue samples from locally and temporarily related wild voles (likely from the same colony or family), one of which was affected by two extra-hepatic tapeworm cysts. The identified tapeworm, *H. taeniaeformis,* is widely abundant globally [30]. Like other tapeworms, this parasite has been documented in a variety of mammals, primarily infecting cats and other feline species [31] (Figure 4A). *H. taeniaeformis* typically infects felines, with rodents serving as the primary intermediate host for the larval form, strobilocercus [32,33].

From a microbiome perspective, it is well known that tapeworms affect the gut microbiome in humans and animals [15,34,35,36]. They produce secretory molecules which affect the gut microbiota [37], and the infestation promotes the production of immunoglobulins (IgG, IgG1, IgG2a, IgG2b, IgG3 and IgM) against gut commensals that correlate with increases or decreases in the feces [15]. Despite this knowledge, little is known about the microbiome features of the cystic structures of tapeworm larva or other migratory parasites and the potential they may have to cause local infections. In our study, the identification of bacteria inside the parasitic peri-hepatic cysts, opens new possibilities for understanding the complicated interplay of viruses, parasites and bacteria in the peritoneal cavity of rodents.

From an ecological perspective, the unanticipated discovery of *Mythimna unipuncta granulovirus* A (MyunGV-A), commonly a lepidopteran-specific baculovirus, in addition to a community of bacteria such as *Lactobacillus acidophilus*, *Enterococcus faecium*, *Bacteroidales* and *Klebsiella variicola*, inside *H. taeniaeformis* cysts in wild voles challenges conventional theories of host-virus specificity and microbial dynamics in ecosystems. Although we did not verify the presence of viruses with cultivation methods or electron microscopy visualization, the presence of MyunGV-A is intriguing because *Baculoviruses*, such as MyunGV-A, use receptors on gut cells of their insect hosts to enable virus entry, reproduction, and dissemination within the insect cells [38,39]. The gastrointestinal system of voles, which is normally a reservoir for a diverse range of commensal bacteria [40,41] could serve as a suitable environment for MyunGV-A survival and a likely replication which we did not visualize [42] or quantify [43]. It is unclear if the virus interacts with the strobilocercus microbiota, potentially aiding the survival of the community within the parasite in the mammalian host. If it does not adapt with replication, another possibility to observe the infection in the mouse could be through dietary acquisition, in which voles consume MyunGV-A-carrying insects and temporarily allow the virus to replicate, until the host clears the viral infection. Although the virus does not reproduce within the vole digestive tract, it is possible that it could survive in the gastrointestinal environment, if the virus symbiosis takes place with bacteria, which has not been reported in MyunGV-A viral laboratory strains. Additionally, the virus and bacteria could enter the systemic circulation via breaks in the mucosal surfaces of the digestive tract of the voles, resulting in broad translocation and colonization throughout the peritoneal cavity. The specificity of these viruses is highlighted by their successful usage as biopesticides [44,45], which is due to their inability to cross species and infect non-target mammals. Therefore, the existence of MyunGV-A within the rodent tapeworm larval cysts needs further examination before considering this presumed co-adaptation in migratory parasites as a strategy for non-traditional viral transmission and survival mechanisms outside the insect gut.

The findings also complement the results from a recent study where parasitic tapeworms of domestic animals in China showed a highly variable virome, but did not identify MyunGV-A in adult parasites [46], which indicates several possibilities and theories for future testing (Figure 4B). Those theories include that adult tapeworm of domestic animals do not carry MyunGV-A or that there are virome differences in China vs. USA, with China not having MyunGV-A, or that MyunGV-A is likely associated with the gut microbiome of wild voles locally in Ohio being also independent of the viral load of adult tapeworms.

In conclusion, although our study is only based on the analysis of three spatially and temporarily related voles, our findings revealed a remarkably reproducible simplified microbial community within the parasitic cysts of wild rodents, which contained gut commensals (*P. distasonis*) and an unexpected insect virus, which thrive reproducibly and independently in spatially-restricted niches, away from the gut, devoid of host-nutrient availability from ingested food or gut ingesta. Metacestodes represent a unique model of bacterial community evasion of the immune system and survival in a host-nutrient-deprived, parasite-acquired environment. This study provides a new perspective on the understanding of bacterial communities in migratory tapeworms and in dysbiosis associated with chronic intestinal diseases where spatially-restricted cavitating micro niches develop and could perpetuate inflammation through symbiotic mechanisms. Such a scenario exists in the case of Crohn’s disease, where we discovered that *P. distasonis* predisposes susceptible hosts to inflammation driven by succinate (*P. distasonis* metabolite) [7] and increases the level of cell vulnerability (cytotoxicity of immune cells) to other bacteria co-habiting in the same micro niche (CavFT) [5,6], namely *Escherichia coli* mice [7,25]. The present study highlights the fact that *P. distasonis* (*Bacteroidota*) and *Enterobacteriaceae* could be highly symbiotic or synergistic to favor their own selection and thrive in confined microniches. Therefore, this observation could help identify revised strategies for the treatment of clinical cysticercosis and the potential benefits of adding antibiotics to patients with cysts of microlesions where *Bacteroidota* and *Enterobacteriaceae* could co-exist.

## Data Availability

All sequence data is available at the NCBI GenBank under BioProject number PRJNA1053337. Edited Sanger DNA sequence was submitted to GenBank accession PP477764.
